# Study on the Properties of Basalt Fiber-Modified Phosphogypsum Planting Concrete

**DOI:** 10.3390/ma18143209

**Published:** 2025-07-08

**Authors:** Weihao Zhang, Xiaoyan Zhou, Menglu Liu, Peng Yuan, Zhao Liu, Chen Shen, Mingwang Hao, Fengchen Zhang, Hongqiang Chu

**Affiliations:** 1 China Yangtze Power Co., Ltd., Yibin 644612, China; zhang_weihao@ctg.com.cn (W.Z.); zhou_xiaoyan@ctg.com.cn (X.Z.); yuan_peng@ctg.com.cn (P.Y.); liu_zhao@ctg.com.cn (Z.L.); 2 College of Civil and Transportation Engineering, Hohai University, Nanjing 210098, China; fczhang@hhu.edu.cn (F.Z.); chq782009@126.com (H.C.); 3 College of Materials Science and Engineering, Hohai University, Nanjing 210098, China; 13291392299@163.com (C.S.); hmw122908054@126.com (M.H.)

**Keywords:** planting concrete, phosphogypsum, basalt fiber, compressive strength, porosity, water retention rate

## Abstract

Planting concrete exhibits notable advantages, including effective reduction of waterborne pollutants, significant ecological restoration capacity, and alignment with principles of green and sustainable development. As a result, it has been increasingly utilized in slope protection and infrastructure construction. In this study, phosphogypsum-based planting concrete was modified using basalt fibers to enhance its mechanical and permeability-related properties. A series of laboratory tests was conducted to evaluate compressive strength, porosity, and sand permeability. The results indicated that the incorporation of basalt fibers effectively improved the compressive strength of the phosphogypsum planting concrete, with longer fibers (18 mm) contributing to a more pronounced enhancement than shorter fibers (6 mm). Moreover, an increase in fiber content led to a gradual decrease in porosity. The addition of basalt fibers also reduced both sand permeability and the water permeability coefficient. Meanwhile, specimens containing 6 mm fibers exhibited a greater reduction in permeability than those with 18 mm fibers. Furthermore, higher fiber content was found to significantly enhance the water retention capacity of the concrete. These findings provide a theoretical basis for the design and optimization of fiber-reinforced planting concrete for ecological engineering applications.

## 1. Introduction

Planting concrete is a multifunctional eco-engineered composite material, which fills the pores with planting substrates to enable plant growth. It is primarily used in slope protection and ecological restoration [1]. Compared to conventional slope protection methods, planting concrete offers superior vegetation survival rates, more effective ecological restoration, and improved cost efficiency [2].

However, ensuring the compatibility of plants with planting concrete remains a significant challenge, particularly regarding strength, porosity, and nutrient provision [3,4,5,6]. Adequate strength is essential for maintaining durability, while porosity plays a crucial role in supporting plant growth within concrete structures. Excessive porosity compromises the mechanical strength of concrete, reducing its protective capacity. Conversely, insufficient porosity hinders root penetration, preventing plants from anchoring into the underlying soil and weakening the bond between the concrete and soil [3]. Furthermore, the continuous hydration process within concrete generates substantial calcium hydroxide. It maintains an alkaline environment with a pH of approximately 13, which is unsuitable for plant growth. Since most plants thrive within a pH range of 6 to 9, it is necessary to apply alkali reduction treatments to planting concrete [7]. Phosphogypsum is used to improve the internal growth environment of planting concrete, providing nutrients for plant growth and promoting the sustainable use of industrial waste residues. However, phosphogypsum planting concrete has limitations, such as low compressive strength and poor water retention.

Fibers are short, discontinuous materials that can be uniformly dispersed in concrete, working synergistically with other constituents to enhance the composite’s properties. They are commonly used in concrete composites to improve tensile and flexural strength, toughness, and overall performance [8,9,10]. Commonly employed fibers for enhancing concrete properties include steel fibers, glass fibers, polypropylene fibers, and basalt fibers. Among these, basalt fibers stand out due to their abundant raw material availability, excellent corrosion resistance, high strength and toughness, and cost-effectiveness. Additionally, their density is comparable to that of concrete, facilitating better bonding and effectively improving the mechanical properties and durability of the concrete matrix [11]. Consequently, basalt fibers have emerged as a promising reinforcement material for concrete composites in various applications.

At present, there have been numerous studies on the effects of basalt fiber content and length on concrete performance. For instance, Yao et al. [12] investigated the protective effects of basalt fibers on concrete under various hydraulic erosion stages. Their findings revealed that basalt fiber-reinforced concrete exhibited, on average, 20% higher erosion resistance than plain concrete under identical conditions, with the improvement becoming more pronounced with extended curing periods. Additionally, under different curing time and water–cement ratios, the compressive strength of basalt fiber-reinforced concrete increased by 10.1% to 17.5%. Chen et al. [13] compared the performance of nine ultra-high-performance concrete (UHPC) mixes containing large basalt fibers with two mixes incorporating steel or polypropylene fibers. They observed that UHPC with 3% large basalt fibers demonstrated more uniform fiber distribution. Compared to counterparts with steel or polypropylene fibers, the UHPC with basalt fibers had superior compressive strength (138.9 MPa), tensile strength (8.14 MPa), and ultimate tensile strain (1714 με). Xie et al. [14] demonstrated that basalt fibers enhance the damage resistance of reactive powder concrete (RPC). Their study on the mechanical properties, energy evolution, and failure characteristics of basalt fiber-reinforced RPC revealed that a 1.0% fiber content significantly improved dynamic compressive strength. However, for the RPC with fiber contents of 1.5% and 2.0%, the increase in compressive strength was limited, and more test pieces showed a decrease in strength. This indicates that there is a threshold for the content of basalt fiber in the active powder concrete. Only by reasonably selecting the content of basalt fiber can the performance of the active powder concrete be improved. Khan et al. [15] studied the compressive and fracture properties of hybrid fiber-reinforced concrete (HyFRC) with basalt fibers of varying lengths (12, 25, 37, and 50 mm) and contents (0.15% and 0.3%). They reported that a fiber length of 25 mm and a content of 0.3% yielded the highest fracture toughness, with longer fibers (>25 mm) showing reduced toughness, though still outperforming plain concrete. Wang et al. [16] evaluated the workability and compressive strength of basalt fiber-reinforced concrete under axial loading, considering five fiber lengths and eight volume contents. They identified optimal fiber lengths of 12–24 mm and a volume content of approximately 0.15%. Ding et al. [17] explored the compressive strength, porosity, and fractal dimension of ecological porous concrete utilizing coarse aggregates ranging from 19 to 26.5 mm. After a 28-day curing period, the concrete exhibited an average compressive strength of 8.76 MPa, a porosity range of 25–30%, predominant pore sizes of 5–7 mm, and fractal dimensions between 1.2 and 1.8. A linear correlation between fractal dimension and porosity was established, elucidating the linkage between pore structure and macro–micro performance. Short fibers fail to provide effective bridging, limiting mechanical enhancements, while excessively long fibers may agglomerate, introducing defects, such as voids and microcracks [18]. From a microstructural perspective, Liang et al. [19] used scanning electron microscopy (SEM) to analyze the role of basalt fibers. They found that basalt fiber incorporation increased the number of micropores while reducing the proportion of larger pores, with finer fibers yielding more significant pore structure optimization [20]. Yu et al. [21] examined the mechanical properties and microstructure of concrete containing 0.20 vol% basalt fiber powder. The experimental results showed that the abrasion resistance of concrete with 0.20 vol% basalt fiber powder was significantly improved. Based on SEM analysis, it was concluded that the addition of basalt fiber powder promoted the development of calcium silicate hydrate (CSH), refined the pore structure of the concrete and promoted its densification. Wang et al. [22] investigated the relationship between pore structure, compressive strength, and permeability of planting recycled concrete (PRC) using industrial CT and image analysis. Compressive strength decreases with increasing porosity, following the Ryshkewitch model. Permeability coefficient increases linearly with porosity, with the power law model providing a better fit than the Kozeny–Carman model.

In summary, basalt fibers significantly enhance the mechanical properties and durability of concrete composites, provided fiber content and length are optimized. These findings underscore the importance of tailoring basalt fiber parameters to specific concrete applications, informing the design of high-performance materials, like phosphogypsum-based planting concrete in this study. As one of the four key fibers prioritized for development in China, basalt fibers offer environmental benefits, high-tensile strength, good elongation at break, and excellent chemical corrosion resistance, making them increasingly utilized in infrastructure, slope protection, and roadbed engineering [23,24]. In this study, basalt fibers with varying dosages and lengths were integrated into phosphogypsum-based planting concrete. A series of performance tests, including compressive strength, porosity, and water retention rate, were conducted to evaluate the performance of basalt fibers in phosphogypsum-based planting concrete. The obtained results can provide technical guidance for the preparation and application of planting concrete.

## 2. Experiment Materials and Method

### 2.1. Experiment Materials

In this study, Conch brand P·O 42.5 ordinary Portland cement produced by Anhui Conch Group Co., Ltd., Wuhu, China, was used. Its physical properties and chemical compositions are provided in Table 1 and Table 2, respectively. Table 3 outlines the main chemical components of phosphogypsum, which was supplied by Anhui Liuguo Chemical Co., Ltd., Tongling, China. Basalt fibers with lengths of 6 mm and 18 mm were utilized as fillers in phosphogypsum planting concrete, and their key properties are summarized in Table 4. The basalt fibers were produced by Sichuan Erun Basalt Fiber Technology Co., Ltd., Chengdu, China.

### 2.2. Specimen Preparation

**Figure 1:**
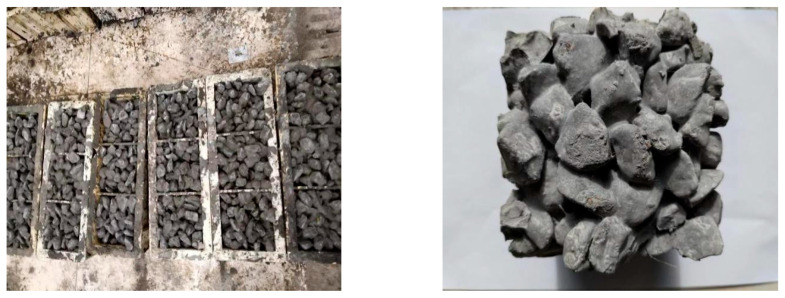
Schematic diagram of the preparation and molding of phosphogypsum planting concrete specimens.

This study adopted a baseline mix proportion with a water-to-binder ratio of 0.3, a designed porosity of 27%, and a phosphogypsum replacement rate of 20% (corresponding to 63 kg/m^3^) to systematically investigate the effects of basalt fiber on the performance of phosphogypsum-based planting concrete. The length (6 mm and 18 mm) and dosage (0%, 0.5%, 1%, and 1.5% by mass of the cement–phosphogypsum binder) of basalt fiber were selected as variables. The selection of a 20% phosphogypsum replacement rate was informed by prior research from our group and supported by literature. Cheng et al. [27] demonstrated that this replacement rate significantly reduces concrete alkalinity, while maintaining adequate compressive strength. Furthermore, Zhang et al. [28] reported that a phosphogypsum dosage of 86 kg/m^3^ yielded compressive strengths of 18.2 MPa at 7 days and 22.6 MPa at 28 days, but higher dosages may compromise long-term durability due to excessive sulfate content. Liu et al. [29] indicated that increased phosphogypsum content elevates trace element levels, leading to toxicity that inhibits seed germination, thus justifying the 20% replacement rate as an optimal choice. For each mix proportion (as shown in Table 5), three specimens with dimensions of 150 mm × 150 mm × 150 mm were prepared and tested. The average value of the three specimens in each group was calculated as the test result to ensure reliability. The prepared phosphogypsum planting concrete specimens are shown in Figure 1.

The concrete specimens were prepared using the paste-coating method. First, cement, supplementary cementitious materials, chemical admixtures, and water were added into a mixing pot sequentially. Coarse aggregate was added when the fresh concrete reached a moderate consistency. After that, the mixture was cast into molds in two equal layers to ensure the fresh concrete distribution uniformly. Each layer was tamped with 20–30 evenly distributed strokes to expel entrapped air. Finally, additional compaction was applied to ensure proper specimen formation.

### 2.3. Experimental Methods

#### 2.3.1. Compressive Strength Test

Following the Standard GB/T 50081-2019 [30], planting concrete specimens with dimensions of 150 mm × 150 mm × 150 mm were prepared. After demolding, the specimens were cured under standard curing conditions (20 ± 1 °C, 95% RH), followed by compressive strength testing at 7 and 28 d.

#### 2.3.2. Porosity Test

Following the Standard CJJ/T253-2016 [31], standard concrete specimens with dimensions of 150 mm × 150 mm × 150 mm were prepared. After demolding, the specimens were cured under standard curing conditions for 7 d, and the mass of a specimen was recorded as *m*_1_. Then, the specimens were placed in a drying chamber and dried at 60 °C for 24 h, and the mass was recorded as *m*_2_. Subsequently, the dried specimens were completely immersed in water for 24 h. When there were no bubbles emerging in the water, the mass of a specimen was recorded as *m*_3_. Based on the above data, the effective porosity *C*_1_ and the total porosity *C*_2_ of the specimens could be calculated using Equations (1) and (2), respectively. The effective porosity refers to the connected porosity of the concrete specimens, and the total porosity is the sum of the connected closed porosity of the specimens.

(1) $$C_{1}=\left(1-\frac{m_{1}{-m}_{3}}{\rho V}\right)\times 100\%$$

(2) $$C_{2}=\left(1-\frac{m_{2}{-m}_{3}}{\rho V}\right)\times 100\%$$

where *C*_1_ is the effective porosity of planting concrete (%); *C*_2_ is the total porosity of planting concrete (%); *V* is the apparent volume of specimen (cm^3^); *ρ* is the density of the water (g/cm^3^); *m*_1_ is the mass of the specimen under standard curing conditions (g); *m*_2_ is the drying quality of the specimen, g; *m*_3_ is the immersed mass of the specimen (g).

#### 2.3.3. Water Retention Rate Test

Following the Standard CJJ/T253-2016 [31], standard concrete specimens with dimensions of 150 mm × 150 mm × 150 mm were prepared. After demolding, the specimens were cured under standard curing conditions for 7 d, and the mass of a specimen was recorded as *m*_1_. Then, the specimens were dried and their mass was recorded as *m*_2_. Subsequently, these dried specimens were completely immersed in water for 24 h. The mass of a specimen was recorded as *m*_3_ when no bubbles emerged in the water. By combining Equations (3) and (4), the natural water absorption rate *A*_1_ and the saturated water absorption rate *A*_2_ of the concrete specimens could be calculated respectively.

(3) $$A_{1}=\left(\frac{m_{1}{-m}_{2}}{m_{2}}\right)\times 100\%$$

(4) $$A_{2}=\left(\frac{m_{3}{-m}_{2}}{m_{2}}\right)\times 100\%$$

where *A*_1_ is the natural water absorption rate of planting concrete (%); *A*_2_ is the saturated water absorption rate of planting concrete (%); *m*_1_ is the quality of the specimen under standard curing conditions (g); *m*_2_ is the drying quality of the specimen (g); *m*_3_ is the immersion quality of the specimen (g).

#### 2.3.4. Sand Permeability Rate Test

According to the Standard JC/T 2557-2020 [32], the sand permeability rate of concrete specimens was tested using standard sand. Initially, the common sand for building was washed to remove impurities and then dried for 24 h. Subsequently, sand particles with diameters ranging from 2.0 to 4.0 mm were sieved for the test. The prepared concrete specimens were fixed. After thorough inspection, they were further fixed on a sieve with an aperture over 4 mm. Subsequently, 150 g of processed sand was evenly spread over them, and a collection tray was positioned underneath to capture the permeating particles. The assembled fixture, sieve, and tray were vibrated for 5 min, and then the weight of the sand accumulated in the tray was measured. The sand permeability rate was determined as the ratio of the collected sand weight to the initial 150 g sample, expressed as a percentage.

#### 2.3.5. Water Permeability Coefficient Test

The specimens were cured for 27 days under standard curing conditions, followed by immersion in water maintained at 20 ± 2 °C for 24 h, and then tested for water permeability coefficient. The physical dimensions of each specimen, including length (*a*), width (*b*), thickness (*L*), and upper surface area (*A*) were measured. Then, the specimens were placed in a water permeability coefficient apparatus with a water level maintained at a constant height of 150 mm. After outflow stabilized, the volume of water discharged within 90 s (*Q*), and hydraulic head difference (H) between the apparatus and the upper surface of the specimen were recorded. The water permeability coefficient was calculated using Equation (5).

(5) $$K=\frac{\mathrm{QL}}{\mathrm{AHt}}$$

where *K* is the water permeability coefficient of specimen (cm/s); *Q* is the water discharge volume within time t seconds (cm^3^); *L* is the thickness of specimen (cm); *A* is the upper surface area of specimen (cm^2^); *H* is the water head difference (cm); *t* is time (t).

#### 2.3.6. Alkalinity Test

Fresh concrete specimens (20 mm × 20 mm × 20 mm) were cured to the specified curing age and transferred to a wide-mouthed flask. After adding 2000 mL deionized water, the bottle was sealed and stored for a designated period. By using a pH meter, the pH value of the pore leachate from specimens was measured to evaluate the alkalinity within the pore.

## 3. Results and Discussion

### 3.1. Compressive Strength Analysis

**Figure 2:**
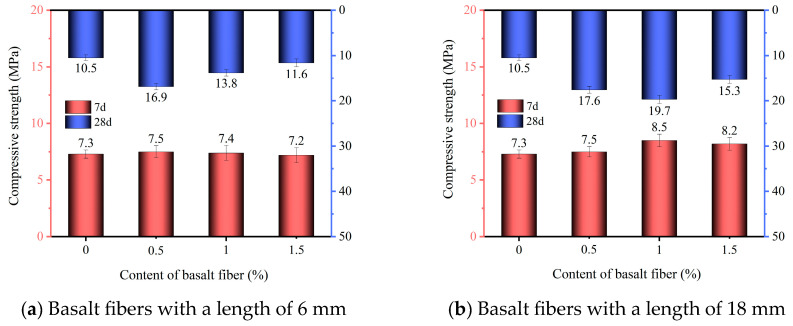
Compressive strength of phosphogypsum planting concrete reinforced with basalt fiber.

The 7-day and 28-day compressive strengths of phosphogypsum-based planting concrete incorporating basalt fibers are presented in Figure 2a and Figure 2b, respectively. These results illustrate the influence of different basalt fiber contents on the compressive strength of the material.

As shown in Figure 2a, 6 mm basalt fibers at 0.5%, 1.0%, and 1.5% content by mass increased the 7-day compressive strength of the specimens to 7.3 MPa, 7.5 MPa, and 7.4 MPa, respectively. Compared with the fiber-free control group (X0-0, 7.2 MPa), these values represent improvements of 1.4%, 4.2%, and 2.8%, respectively. Similarly, compared with the X0-0 control group, the 28-day strength increased from 10.5 MPa to 16.9, 13.8, and 11.6 MPa at 0.5%, 1.0%, and 1.5% fiber content, which represent increases of 61.0%, 31.4%, and 10.5%, respectively. As shown in Figure 2b, with 18 mm basalt fibers at 0.5%, 1.0%, and 1.5% content, the 7-day compressive strength of the specimens was 7.2 MPa to 7.5, 8.5, and 8.2 MPa, corresponding to increases of 4.2%, 18.1%, and 13.9%, respectively. The 28-day strength increased from 10.5 MPa to 17.6, 19.7, and 15.2 MPa, representing gains of 67.6%, 87.6%, and 44.8%, respectively. According to JC/T 2557-2020 [32], planting concrete should attain minimum compressive strengths of 3 MPa at 7 d and 10 MPa at 28 d. All fiber-reinforced groups met or exceeded these requirements, confirming the suitability for engineering applications. The 87.6% increase in compressive strength at 1.0% 18 mm fiber is significant. To contextualize this result, comparisons with similar studies involving other fibers or waste-based concretes are necessary. Mojtaba et al. [33] investigated the compressive strength enhancement of concrete specimens with polyolefin and polypropylene fibers. Their results showed that, compared to plain concrete, specimens with polypropylene fibers and polyolefin fibers exhibited average compressive strength increases of approximately 31.47% and 38.36%, respectively. Bassam A. et al. [34] evaluated the mechanical properties of concrete with different steel fiber shapes and contents. They found that hooked and corrugated steel fibers outperformed straight fibers. Compared with concrete with straight fibers of the same volume, the compressive strength of concrete composed of 2.5% hooked fibers and corrugated fibers increased by 50% and 65%, respectively. In contrast, the 87.6% improvement observed in our study with 18 mm basalt fibers is substantially higher, likely due to the superior tensile strength and elastic modulus of basalt fibers, as well as their ability to form a robust spatial interlocking network within the phosphogypsum-based matrix [35].

These results indicate that basalt fibers enhance the compressive strength of phosphogypsum planting concrete. This improvement of compressive strength is attributed to the formation of a spatial interlocking fiber network within the cement matrix, which effectively bridges microcracks and imparts multidirectional reinforcement [23,36]. Such a network provides lateral confinement during loading, thereby the compressive strength of the specimen is improved [37]. Furthermore, basalt fibers inherently possess high tensile strength, good elongation at break, and a high elastic modulus. Therefore, the incorporation of basalt fibers can enhance the compressive strength of phosphogypsum-based planting concrete [38].

As seen from Figure 2, for both 6 mm and 18 mm fiber types, the 7-day compressive strength of the specimens increased with fiber content up to an optimal dosage, beyond which a reduction was observed, which is consistent with the findings of Wang et al. [39]. Meanwhile, the 7-day compressive strength of phosphogypsum-based planting concrete incorporating 6 mm basalt fibers reached its maximum value at a fiber content of 0.5%. For concrete with 18 mm basalt fibers, the highest 7-day compressive strength was observed at a fiber content of 1.0%. This phenomenon is attributed to the tendency of excessive basalt fibers to agglomerate, which led to stress concentration in certain regions of the specimen. Moreover, the 7-day compressive strength of phosphogypsum planting concrete incorporating 18 mm basalt fibers was consistently higher than that of specimens with 6 mm fibers at the same dosage. This is attributed to the fact that 18 mm basalt fibers are more capable of forming a uniform and stable spatial load-transferring fiber network compared to 6 mm fibers. Compared with the short fibers, longer fibers exhibit a lower warping factor and better shrinkage resistance of longer fibers [40]. Festugato et al. [41] also pointed out that increasing the fiber length contributes to enhancing the mechanical performance of the fiber itself. Consequently, longer fibers not only improve the overall integrity of the specimen but also promote more uniform stress distribution, which results in a more noticeable increase in compressive strength.

### 3.2. Porosity Analysis

**Figure 3:**
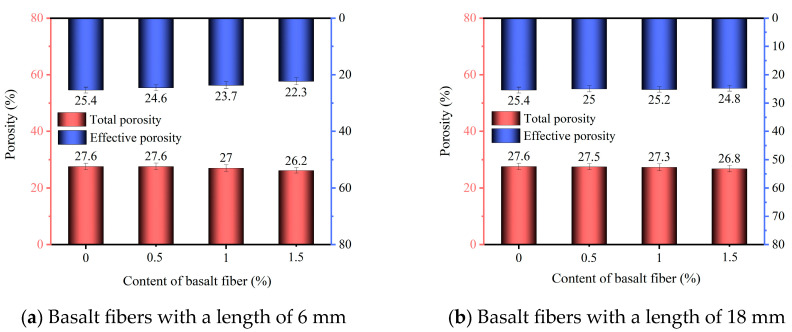
Porosity of phosphogypsum planting concrete incorporating basalt fibers.

The effective and total porosities of phosphogypsum planting concrete incorporating 6 mm and 18 mm basalt fibers are illustrated in Figure 3a and Figure 3b, respectively. When no basalt fiber was added, the total porosity of the concrete was 27.6%. With the addition of 6 mm basalt fibers at the content of 0.5%, 1.0%, and 1.5%, the total porosity of the specimens decreased to 27.6%, 27.0%, and 26.2%. Compared to the control group, corresponding to reductions of 0%, 2.2%, and 5.1%, respectively. For 18 mm basalt fibers, the total porosity was reduced to 27.5%, 27.3%, and 26.8%, representing decreases of 0.3%, 1.1%, and 2.9%, respectively.

In the control group (X0-0), the effective porosity was 25.4%. When 6 mm basalt fibers were added at 0.5%, 1.0%, and 1.5% content, the effective porosity decreased to 24.6%, 23.7%, and 22.3%, which were 3.1%, 6.7%, and 12.2% lower than the control, respectively. With 18 mm basalt fibers, the effective porosity was reduced to 25.2%, 25.0%, and 24.8%, corresponding to decreases of 0.8%, 1.6%, and 2.4%, respectively.

Overall, Figure 3 indicates that both the total and effective porosities decrease with increasing basalt fiber content. This is mainly because the added fibers occupy internal voids within the concrete matrix [42]. Under the same fiber content, the total and effective porosities of concrete with 18 mm basalt fibers were higher than the specimens with 6 mm fibers, which is attributed to the more uniform dispersion of the shorter 6 mm fibers, which can better fill voids and thereby reduce effective porosity [24].

In addition, with 6 mm basalt fibers at 0.5%, 1.0%, and 1.5% content, the effective porosity of the phosphogypsum planting concrete was 2.2%, 3.0%, and 3.3% lower than its corresponding total porosity, respectively. When 18 mm basalt fibers were added at the content of 0.5%, 1.0%, and 1.5%, the differences between total and effective porosity were 2.5%, 2.1%, and 2.0%, respectively. This indicates that the proportion of closed pores decreased with increasing content of 18 mm fibers. The underlying reason is that shorter basalt fibers with lower dosage exhibit higher degrees of freedom and are less prone to agglomeration [36]. Therefore, compared to longer fibers, shorter basalt fibers are more effective at sealing internal pores within the specimen.

### 3.3. Sand-Passing Ratio Analysis

**Figure 4:**
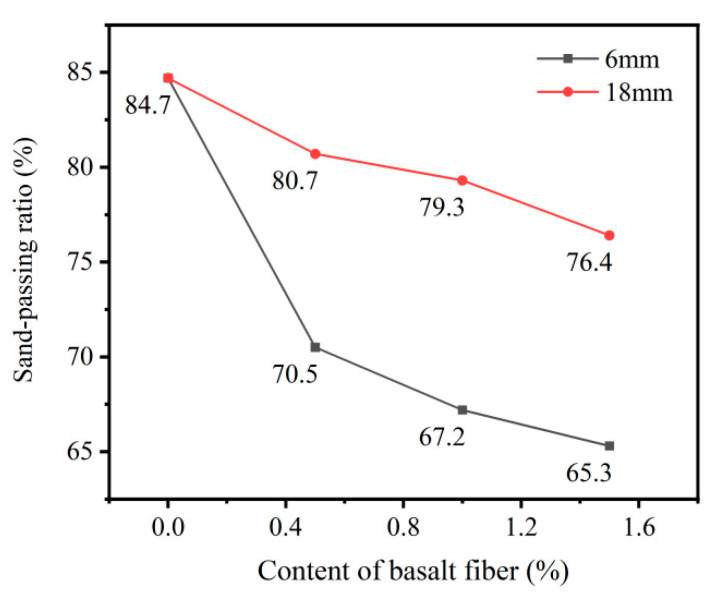
Sand-passing ratio of phosphogypsum planting concrete with basalt fiber.

The sand-passing ratios of basalt fiber-reinforced phosphogypsum planting concrete are presented in Figure 4. In the control specimen without basalt fibers, the sand-passing ratio was 84.7%. With 6 mm basalt fibers at 0.5%, 1.0%, and 1.5% content, the ratios dropped to 70.5%, 67.2%, and 65.7%, respectively, corresponding to reductions of 16.8%, 20.6%, and 22.4%. For 18 mm basalt fibers, the values decreased to 80.7%, 79.3%, and 76.4% at the same dosages, with reductions of 4.7%, 6.4%, and 9.8%, respectively.

The sand-passing ratio of the specimens exhibited a downward trend with increasing basalt fiber content. This is closely associated with the concurrent reductions in both total and effective porosity. Additionally, the rise in fiber content decreased the size and connectivity of continuous pores within the matrix, thereby lowering the sand-passing capacity.

Notably, the reduction in sand-passing ratio was more pronounced in specimens with 6 mm fibers than those with 18 mm fibers. This was attributed to the ability of the longer 18 mm basalt fibers to form a more effective spatial network within the fresh concrete, which reduces the likelihood of slurry sedimentation [23,36,43].

As stipulated in JC/T 2557-2020 [32], the sand-passing ratio—used to characterize the vegetative pore structure—must not fall below 50%. All specimens with fiber content ≤1.5% met this requirement, with sand-passing ratios exceeding 65% and 76% for 6 mm and 18 mm fibers, respectively. This confirms that the effective porosity of phosphogypsum planting concrete incorporating both 6 mm and 18 mm basalt fibers meets the requirements specified by the relevant standards.

### 3.4. Permeability Coefficient Analysis

**Figure 5:**
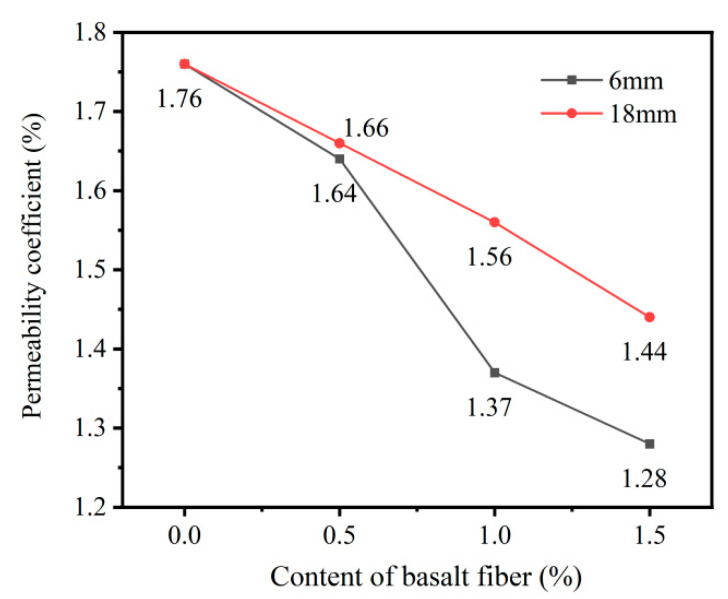
Permeability coefficient of phosphogypsum planting concrete with basalt fiber.

The water permeability coefficients of basalt fiber-reinforced phosphogypsum vegetation concrete are shown in Figure 5. The coefficient for the control specimen without basalt fibers was 1.76 cm/s. With 6 mm basalt fibers at 0.5%, 1.0%, and 1.5% content, the permeability of the specimens decreased to 1.64, 1.37, and 1.28 cm/s, corresponding to reductions of 6.8%, 22.2%, and 27.3%, respectively. For 18 mm fibers, the permeability dropped to 1.66, 1.56, and 1.44 cm/s at the same dosages, with reductions of 5.7%, 11.4%, and 18.2%, respectively.

Furthermore, as shown in Figure 5, the permeability coefficient of phosphogypsum planting concrete decreases with increasing basalt fiber content. This reduction was attributed to the fact that basalt fibers occupy internal pores within the concrete matrix, thereby gradually reducing both total and effective porosity. Additionally, the spatial network formed by the basalt fibers increases the internal flow resistance, further contributing to the decrease in permeability. Moreover, compared to specimens incorporating 18 mm basalt fibers, those specimens with 6 mm fibers exhibited a more significant reduction in permeability coefficient. This trend is consistent with the variation observed in the sand-passing ratio.

### 3.5. Water Retention Analysis

**Figure 6:**
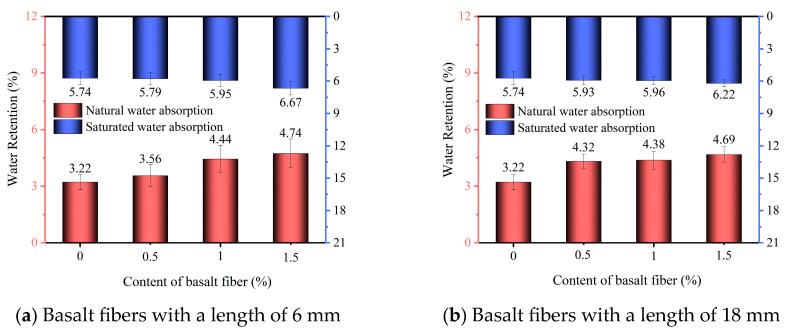
Water absorption of phosphogypsum planting concrete incorporating basalt fibers.

The natural and saturated water absorption capacities of the specimens with 6 mm and 18 mm basalt fibers are presented in Figure 6a and Figure 6b, respectively.

As illustrated in Figure 6, both natural and saturated water absorption increased with higher fiber dosages. At 1.5% dosages of 6 mm fiber, the natural absorption reached 4.74% and saturated absorption 6.67%, increasing by 47.2% and 16.2% compared to the control group. For the 18 mm fiber group, natural and saturated absorption values were 4.69% and 6.22%, respectively—corresponding to increases of 45.6% and 8.3%.

The water retention capability of planting concrete depends mainly on its internal pore structure and the water absorption characteristics of its components. In general, matrices with a higher proportion of fine, interconnected pores exhibit greater water-holding capacity, while those with more coarse pores retain less moisture. As basalt fiber content increases, the proportion of fine pores within the matrix also increases [44]. Therefore, the water retention capacity of the planting concrete was enhanced, leading to increased natural and saturated water absorption.

### 3.6. Alkalinity Analysis

**Figure 7:**
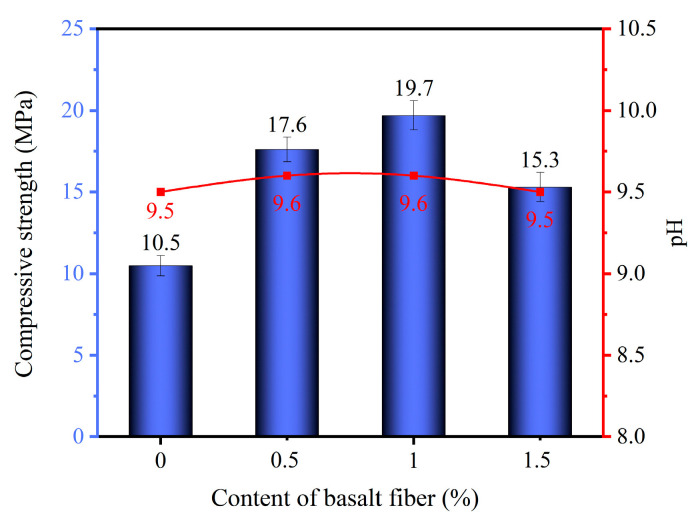
Alkalinity and compressive strength of phosphogypsum planting concrete with 18 mm basalt fiber.

The alkalinity of phosphogypsum planting concrete reinforced with 18 mm basalt fibers was tested, and the results are shown in Figure 7. With increasing fiber content, both compressive strength and pH values initially increased and then decreased. Specifically, at fiber contents of 0.0%, 0.5%, 1.0%, and 1.5%, the corresponding pH values were 9.5, 9.6, 9.6, and 9.5, respectively.

Compared with the changes in compressive strength, the pH variation was relatively minor. This minimal pH change is significant for the suitability of the planting concrete for plant growth. As noted in the introduction, most plants thrive in a pH range of 6 to 9, while conventional concrete often maintains a highly alkaline environment (pH ≈ 13), which is detrimental to vegetation. The observed pH values of approximately 9.5–9.6 in the basalt fiber-reinforced phosphogypsum planting concrete fall within or very close to the optimal range for plant growth, indicating that the material provides a favorable chemical environment for vegetation. This is likely due to the incorporation of phosphogypsum, which helps neutralize the alkalinity of the cement matrix, combined with the negligible influence of basalt fibers on the internal alkaline environment. Therefore, the incorporation of basalt fibers not only enhances the mechanical and structural properties of the planting concrete but also maintains its compatibility with plant growth requirements.

## 4. Conclusions

The incorporation of basalt fibers into phosphogypsum-based planting concrete significantly enhances its performance across multiple properties, with variability observed depending on fiber length and dosage. The specific findings, including the variability of measured indicators, are as follows:

1. The incorporation of basalt fibers significantly enhanced the compressive strength of phosphogypsum planting concrete. Specimens with 18 mm basalt fibers exhibited higher compressive strength than those with 6 mm fibers at the same dosage. The maximum 28-day compressive strength of the specimens with 18 mm basalt fibers reached 19.7 MPa at a fiber content of 1.0%, representing an 87.6% increase compared to fiber-free concrete.
2. Both the total and effective porosity of phosphogypsum planting concrete decreased with increasing basalt fiber content. For 6 mm fibers, total porosity reduced from 27.6% (control) to 26.2% at 1.5% fiber content, while for 18 mm fibers, the reduction was less pronounced, reaching 26.5% at the same dosage. The variability in porosity reduction highlights the superior pore-filling effect of shorter (6 mm) fibers compared to longer (18 mm) fibers, attributed to reduced agglomeration tendencies of shorter fibers.
3. The sand permeability rate and water permeability coefficient of phosphogypsum planting concrete decreased while the basalt fiber content increased. For 6 mm fibers, the sand permeability rate dropped by 16.8% to 22.4% (from 84.7% to 65.7%) and the water permeability coefficient by 6.8% to 27.3% (from 1.76 cm/s to 1.28 cm/s) at 0.5% to 1.5% fiber content. For 18 mm fibers, reductions were smaller, with sand permeability decreasing by 4.7% to 9.8% (to 76.4%) and water permeability by 5.7% to 18.2% (to 1.44 cm/s). The variability indicates that 6 mm fibers are more effective in reducing permeability due to better distribution within the matrix.
4. Both the natural and saturated water retention rates of phosphogypsum planting concrete increased with higher basalt fiber content. For 6 mm fibers at 1.5% content, natural water absorption increased by 47.2% (to 4.74%) and saturated absorption by 16.2% (to 6.67%) compared to the control. For 18 mm fibers, increases were slightly lower at 45.6% (to 4.69%) and 8.3% (to 6.22%). The variability in water retention improvement underscores the role of finer pore structures formed by higher fiber dosages, with 6 mm fibers showing a slightly greater effect.
5. The incorporation of basalt fibers had a minimal impact on the alkalinity of the concrete, with pH values ranging from 9.5 to 9.6 across all fiber contents. This low variability suggests that basalt fibers primarily influence the mechanical and physical properties rather than the chemical environment of the concrete.

This study’s findings underscore the potential of basalt fiber-reinforced phosphogypsum-based planting concrete as a sustainable material for ecological engineering applications. By integrating industrial by-products, like phosphogypsum, with cost-effective basalt fibers, this research promotes waste valorization and reduces the environmental impact of concrete production. The optimized material design offers practical guidance for engineers to develop high-performance planting concrete that balances structural integrity with plant compatibility, contributing to the advancement of green infrastructure solutions.

## Figures and Tables

**Table 1:** Performance indicators of cement.

Fineness (m^2^/kg)	Soundness	Setting Time (min)		Flexural Strength (MPa)		Compressive Strength (MPa)	
		Initial	Final	3 d	28 d	3 d	28 d
360	Qualified	140	260	5.7	8.1	28.9	46.9

**Table 2:** Major chemical composition of cement (wt %).

SiO_2_	Al_2_O_3_	Fe_2_O_3_	CaO	MgO	SO_3_	LOI
21.49	4.89	4.12	62.53	2.22	1.56	3.19

**Table 3:** Major chemical composition of phosphogypsum (wt %).

SO_3_	CaO	SiO_2_	Al_2_O_3_	P_2_O_5_	Fe_2_O_3_	K_2_O	MgO	Na_2_O
43.47	28.79	4.72	0.88	0.62	0.36	0.26	0.23	0.20

**Table 4:** Basic performance parameters of basalt fibers [25,26].

Diameter (μm)	Density (kg/m^3^)	Tensile Strength (MPa)	Elastic Modulus (MPa)
13~14	2600	2500~2900	72~85

**Table 5:** Mix proportions of phosphogypsum planting concrete with basalt fiber addition.

Mixtures	Fiber Length (mm)	Fiber Dosage (%)	Basalt Fiber (kg/m^3^)	Coarse Aggregate (kg/m^3^)	Cement (kg/m^3^)	Phosphogypsum (kg/m^3^)	Water (kg/m^3^)	Admixture (kg/m^3^)
X0-0	0	0	0	1470	252	63	95	9.5
X0.5-6	6	0.5	1.6	1470	252	63	95	9.5
X1-6	6	1	3.2	1470	252	63	95	9.5
X1.5-6	6	1.5	4.8	1470	252	63	95	9.5
X0.5-18	18	0.5	1.6	1470	252	63	95	9.5
X1-18	18	1	3.2	1470	252	63	95	9.5
X1.5-18	18	1.5	4.8	1470	252	63	95	9.5

## Data Availability

The original contributions presented in this study are included in the article. Further inquiries can be directed to the corresponding author.
